# Characterisation of breakfast, lunch, dinner and snacks in the Japanese context: an exploratory cross-sectional analysis

**DOI:** 10.1017/S1368980020004310

**Published:** 2022-03

**Authors:** Kentaro Murakami, Nana Shinozaki, M Barbara E Livingstone, Aya Fujiwara, Keiko Asakura, Shizuko Masayasu, Satoshi Sasaki

**Affiliations:** 1 Department of Social and Preventive Epidemiology, School of Public Health, University of Tokyo, Tokyo 113-0033, Japan; 2 Nutrition Innovation Centre for Food and Health (NICHE), School of Biomedical Sciences, Ulster University, Coleraine, UK; 3 Department of Nutritional Epidemiology and Shokuiku, National Institute of Biomedical Innovation, Health and Nutrition, Tokyo, Japan; 4 School of Medicine, Toho University, Tokyo, Japan; 5 Ikurien-naka, Ibaraki, Japan

**Keywords:** Meal type, Healthy Eating Index-2015, Nutrient-Rich Food Index 9.3, Nutrient density, Japan

## Abstract

**Objective:**

To characterise different meal types by examining the contribution of specific meals to the total intakes and the nutritional quality of each meal.

**Design:**

A cross-sectional analysis was conducted based on dietary data collected using 4-d dietary record. Diet quality was assessed by the Healthy Eating Index-2015 and Nutrient-Rich Food Index 9.3.

**Setting:**

Japan.

**Participants:**

Adults aged 20–81 years (*n* 639).

**Results:**

Diet quality was, on average, highest for dinner, followed, in order, by lunch, breakfast and snacks. Breakfast, lunch, dinner and snacks, on average, accounted for 21 %, 32 %, 40 % and 11 % of total energy intake, respectively. For many nutrients, the percentage contribution to total intake did not vary within each meal, broadly in line with that for energy: 18–24 % for breakfast, 26–35 % for lunch, 35–49 % for dinner and 4–15 % for snacks. However, intakes of many foods largely depended on one meal type. The foods mainly eaten at dinner were potatoes, pulses, total vegetables, fish, meat and alcoholic beverages (52–70 %), in contrast to noodles (58 %) at lunch and bread (71 %) and dairy products (50 %) at breakfast. The foods mainly eaten at snacks were confectioneries (79 %) and sugar-sweetened beverages (52 %). Conversely, rice and eggs were more evenly distributed across three main meals (19–41 % and 30–38 %, respectively), while fruit and non-energetic beverages were more evenly distributed across all meal types (17–30 % and 19–35 %, respectively).

**Conclusions:**

These findings provide the background information on each meal type in Japanese and may help inform the development of meal-based guidelines and public health messages.

Investigation of dietary intake is generally performed in terms of the daily intake of individual foods/food groups and nutrients^(1)^. However, studying dietary intake at the meal level (i.e. breakfast, lunch, dinner and snack) may be more pertinent than overall dietary intake, given that all foods are not always consumed proportionately at each meal but rather when eating, people mainly choose to combine foods in each of the meal types, resulting in meal-specific food combinations^(2–4)^. Unfortunately, data on how different meal types differ in their contribution to the total intake of foods and nutrients are limited, both internationally^(5–9)^ and specifically in the Japanese context. This kind of information would be helpful for formulating meal-based dietary guidelines and public health messages as well as for developing effective intervention strategies for healthy eating.

Mainly because of their possible contribution to a low prevalence of coronary artery disease and long life expectancy^(10,11)^, much attention has focused on the characteristics of Japanese dietary habits, which are somewhat different from those in Western countries^(12)^. Typically, the Japanese diet includes a high consumption of refined grains, soyabean products, seaweeds, vegetables, fish and green tea and a low consumption of whole grains, nuts, processed meat and sugar-sweetened beverages^(13,14)^. At the nutrient level, Japanese diets are high in Na^(15,16)^ but low in saturated fats^(17,18)^ and added sugars^(18,19)^. Furthermore, Japanese diets are high in dietary glycaemic index and glycaemic load^(20)^ but low in dietary energy density^(21)^.

Japanese meal patterns typically consist of a staple food (mainly rice), a main dish (mainly protein-rich foods) and a side dish (mainly vegetables)^(22)^. Furthermore, the proportion of daily energy intake (EI) consumed at breakfast, lunch, dinner and snacks is, on average, 23, 30, 40 and 8 %, respectively, in Japan^(23)^, while the ranges of corresponding values are 9–20 % (median 16 %), 16–45 % (median 25 %), 24–40 % (median 32 %) and 10–34 % (median 26 %), respectively, in the USA^(24)^ and ten European countries participating in the European Prospective Investigation into Cancer and Nutrition calibration study^(25)^. Additionally, recent analyses conducted in the UK^(26)^, the USA^(27)^, Australia^(28)^ and Japan^(29)^ have shown that the associations of frequency of meals (sum of breakfast, lunch and dinner) and snacks with overall diet quality differ among populations, warranting thorough investigation on food and nutrient profiles of each meal type within a specific population.

In the present cross-sectional study, we characterised breakfast, lunch, dinner and snacks in the Japanese context, by examining how each meal contributes to intakes of foods and nutrients, as well as the nutritional quality of each meal. We also examined the associations of overall diet quality with food and nutrient intakes from each of the different meal types.

## Methods

### Data source and analytic sample

The present cross-sectional analysis was based on two independent data sets collected using the similar procedure but at different time periods, that is in 2003 and 2013. As details of both surveys have been provided elsewhere^(16,30–33)^, only a brief description is given here. The 2003 survey was conducted among apparently healthy women and their cohabitating spouses in four geographically diverse areas in Japan: Osaka (urban), Okinawa (urban island), Nagano (rural inland) and Tottori (rural coastal)^(31,32)^. Our recruitment strategy was such that each 10-year age category, namely 30–39, 40–49, 50–59 and 60–69 years, included eight women for each area (without consideration of age of men), resulting in 256 participants. The 2013 survey was conducted among apparently healthy men and women aged 20–69 years working in welfare facilities (and, in some occasions, their neighbours and acquaintances in the over 60 years) in twenty study areas consisting of twenty-three (out of forty-seven) prefectures^(16,33)^. In the recruitment process, each of the areas included two men and two women from each of the five 10-year age groups (20–29, 30–39, 40–49, 50–59 and 60–69 years), resulting in 400 participants. Participation of one individual per household was permitted.

In total, 642 participants (*n* 250 in 2003 and 392 in 2013) provided dietary data for the present analysis. In both surveys, recruitment was conducted until the planned number of participants in each of sex and age groups was enrolled. Unfortunately, the number of potential participants invited was not formally recorded, and thus, the participation rate could not be calculated. After excluding three participants with missing information on the variables of interest, the present analysis was based on 639 individuals. None of the sample was a dietitian, had an experience with dietary counselling from a medical doctor or dietitian or had history of hospitalisation for diabetes education.

### Dietary assessment

Dietary data were collected by a four-non-consecutive day weighed dietary record during the winter season (February and March) in both surveys^(30,33)^. Each recording period comprised three week days (Monday to Friday) and one weekend day (Saturday or Sunday) in the 2003 survey and three working days and one non-working day in the 2013 survey. Each of the recording days was (non-randomly) allocated within approximately 2 weeks by research dietitians. In the latter survey, night shift-working days and days before and after a night shift work were avoided as recording days. Each participant was issued recording sheets and a digital scale (KD-173; Tanita in 2003 and KD-812WH; Tanita in 2013). After receiving written and verbal instructions by a research dietitian, as well as an example of a completed diary sheet, each participant was requested to document and weigh all items eaten or drunk, both in and out of the home, on each of the recording days. On occasions when weighing was problematic (e.g. dining out), they were instructed to document as much information as possible, including the brand name of the food and the consumed portion size (based on typical household measures), as well as the details of leftovers.

The recording sheets for each survey day were submitted directly to the research dietitian after the survey was completed, who then reviewed the forms and, whenever necessary, sought additional information or modification of the record via telephone or in person. All the collected records were then reviewed by research dietitians at each local centre and again at the study centre. As requested in the study protocol, portion sizes estimated using household measures were converted into weights and individual food items were coded based on the Standard Tables of Food Composition in Japan^(34)^. Estimates of intakes of fifteen selected food groups were then calculated for each individual; grouping of foods was done based on similarities in nutrient profile or culinary use of the foods, as shown elsewhere^(9)^. Estimated intakes of energy and selected nutrients for each individual were calculated based on the intakes of food items and their nutrient contents. Added sugar intake, defined as sugars and syrups added to food during processing or preparation, excluding naturally occurring sugars in foods, was also calculated based on a recently compiled comprehensive composition database^(19)^.

### Assessment of diet quality

As measures of diet quality, the Healthy Eating Index 2015 (HEI-2015)^(35-37)^ and Nutrient-Rich Food Index 9.3 (NRF9.3)^(38–41)^ were calculated. The HEI-2015 is a 100-point scale to assess compliance with the 2015–2020 Dietary Guidelines for Americans^(42)^, with a higher score indicating a better quality of overall diet. The HEI-2015 consists of nine adequacy components (total fruits, whole fruits, total vegetables, greens and beans, whole grains, dairy products, total protein foods, seafood and plant proteins, and fatty acids as the ratio of the sum of PUFA and MUFA to SFA) and four moderation components (refined grains, Na, added sugars and saturated fats). We calculated the HEI-2015 component and total scores based on energy-adjusted values of overall dietary intake, namely amount per 1000 kcal of energy or percentage of energy, except for fatty acids^(32)^.

The NRF9.3 is a composite measure of the nutrient density of the total diet, calculated as the sum of the percentage of reference daily values for nine qualifying nutrients, namely protein, dietary fibre, vitamin A, vitamin C, vitamin D, Ca, Fe, K and Mg, minus the sum of the percentage of reference daily values for three disqualifying nutrients, namely added sugars, saturated fats and Na. Reference daily values were determined for sex and age categories, based on the Dietary Reference Intakes for Japanese, 2015^(43)^, except for added sugars, for which the conditional recommendation advocated by the WHO (i.e. upper limit of 5 % of energy)^(44)^ was used because of the lack of a recommended value for added sugars in Japan, as well as their low intake levels^(19)^. We calculated the NRF9.3 component and total scores based on the overall daily intake of each nutrient for each participant, which was adjusted for EI by the density method and then normalised for the sex- and age-specific Estimated Energy Requirement for a moderate level of physical activity (from the Dietary Reference Intakes for Japanese, 2015^(43)^) and expressed as a percentage of the reference daily values^(32)^. Higher NRF9.3 scores indicated a better quality of the overall diet.

Rationale for the choice of these two diet quality measures primarily developed for Americans but not for Japanese was as follows. First, in our recent systematic review of Japanese studies which obtained dietary patterns using principal component analysis, we found that those food groups which contributed to dietary patterns termed healthy (fruits, vegetables, potatoes, mushrooms, seaweeds and pulses) are at least partly similar to those often observed in Western countries (fruits, vegetables including mushrooms, poultry, fish, low-fat dairy products, legumes and whole grains)^(12)^. Further, our recent analysis based on the Japanese National Health and Nutrition Survey supports the efficacy of these measures in assessing the overall diet quality of Japanese: a higher total score in the HEI-2015 and NRF9.3 was associated with favourable patterns of overall diet, including higher intakes of dietary fibre and key vitamins and minerals and lower intakes of saturated fats, added sugars and Na^(18)^.

### Definition of each meal type

The food diary sheet used was based on a typical Japanese eating pattern, comprising breakfast, lunch, dinner and snacks, which were prescribed in the diary^(31)^. During the diet recording, participants were asked to report the clock time when a food or beverage was consumed (both start and finish times). In this study, eating occasions were defined as any separate intake occasion with a discrete start clock time and name, except for eating occasions consisting of water only (tap and mineral water), which were excluded^(45)^. Consequently, all items reported in an eating occasion were given the same clock time and eating occasion name in the food diary.

Each of the eating occasions was categorised into breakfast, lunch, dinner or snacks based on the section in the food diary in which it was recorded, except for the following two situations. The first is multiple entries of eating occasions (with different times) into a section of breakfast, lunch or dinner (only 10 cases). For this, the first eating occasion was considered the main meal (breakfast, lunch or dinner) and the subsequent eating occasions were considered snacks. The second is ≥2 different types of eating occasions recorded within the overlapping time period (243 cases), in which case each of the overlapping eating occasions was combined and counted as a single eating occasion. Where a participant recorded a main meal (breakfast, lunch or dinner) and a snack within the overlapping period, we considered this eating occasion a meal, unless the participant had already recorded that same meal earlier in the day, in which case this eating occasion was considered a snack. It should be noted that the definition of each meal type used is generally consistent with the previous research^(5–9)^.

### Assessment of other variables

Body height was measured without shoes to the nearest 0·1 cm. Body weight was measured in light clothing to the nearest 0·1 kg. BMI (kg/m^2^) was calculated as body weight (kg) divided by the square of body height (m), based on which, weight status was grouped into three categories of underweight (<18·5), normal weight (≥18·5 to <25·0) and overweight (≥25·0)^(46)^. Misreporting of EI was evaluated on the basis of the ratio of EI:BMR (Goldberg’s cut-off)^(47)^. BMR was estimated according to an equation specifically developed for Japanese on the basis of body height and weight, age and sex^(48,49)^. Assuming a physical activity level for a sedentary lifestyle (i.e. 1·55) for all participants (because of a lack of an objective measure of physical activity), underreporting, plausible reporting and overreporting were defined as having EI:BMR of <1·02, ≥1·02 to <2·35 and ≥2·35, respectively^(47)^.

### Statistical analysis

All statistical analyses were performed using SAS statistical software (version 9.4; SAS Institute Inc.). All reported *P* values are two-tailed, and *P* values < 0·05 were considered statistically significant. For all dietary variables, mean daily values over 4 d were used in the analysis to minimise day-to-day variations in dietary intake and a value of zero was assigned for non-consumers. Descriptive statistics of diet quality scores and intakes of energy, nutrients and food groups for total diet and for each meal type are presented as mean and standard deviations. Pearson correlation coefficients among diet quality scores were calculated. Differences in diet quality scores between sex, between survey year and across categories of age (<40, 40–59 and ≥60 years), weight status and dietary reporting status were examined on the basis of independent *t* test or ANOVA followed by a Bonferroni’s *post hoc* test. For each of the dietary variables (energy, nutrients and food groups), the percentage contribution to total intake was calculated for each meal type. Associations between overall diet quality (tertile category of total scores of HEI-2015 and NRF9.3 of total diet) and intakes of energy, nutrients and food groups from each meal type were examined using the general linear model, with adjustment for sex, age group, weight status, dietary reporting status and survey year. Analyses were repeated after stratified by age (by median), sex or survey year, which provided the findings generally similar to those observed in the entire sample (data not shown). The present report, therefore, presents the results for the entire sample. Power calculations were not performed because this study is a secondary analysis of existing data with an exploratory nature.

## Results

The present analysis included 639 Japanese adults with a mean age of 47·1 (sd 13·2) years and a mean BMI of 23·1 (sd 3·4) kg/m^2^. The percentage of participants who reported consumption of breakfast, lunch and dinner on all four dietary recording days was high (88·0, 92·3 and 96·9 %, respectively; see online supplementary material, Supplemental Table 1). In contrast, the prevalence of no consumption of each of these meals on all 4 d was very low (0·8 % for breakfast, 0·5 % for lunch and 0 % for dinner). The percentage of participants who reported consumption of at least one snack on all four dietary recording days was 59·9 %, while the prevalence of no snack consumption on all 4 d was 7·2 %. Mean clock time for the start of breakfast (*n* 634), lunch (*n* 636) and dinner (*n* 639) was 07.28 (sd 00.46), 12.32 (sd 00.32) and 19.25 (sd 00.56) hours, respectively. The daily snack frequency ranged from 0 to 8, with a mean of 1·8 (sd 1·3), while the daily total eating frequency ranged from 1·5 to 11, with a mean of 4·7 (sd 1·3).

Diet quality as assessed by the HEI-2015 and NRF9.3 was, on average, highest for dinner, followed, in order, by lunch, breakfast and snacks (Table 1). As theoretically expected, the quality of each meal type was positively correlated with that of the total diet (Pearson correlation: 0·29–0·65 for HEI-2015 and 0·34–0·70 for NRF9.3). Nevertheless, the correlation between the four meal types was relatively weak (Pearson correlation: 0·09–0·33 for HEI-2015 and 0·15–0·25 for NRF9.3).

**Table 1:** Descriptive statistics of total scores of the Healthy Eating Index-2015 (HEI-2015) and Nutrient-Rich Food Index 9.3 (NRF9.3) in 639 Japanese adults aged 20–81 years

	Mean	sd	Minimum	Q25	Median	Q75	Maximum	Pearson correlation				
								Total diet	Breakfast	Lunch	Dinner	Snacks
HEI-2015*												
Total diet	52·1	7·4	22·1	47·1	51·3	56·9	77·2	1·00				
Breakfast	45·0	12·5	0	37·9	45·2	53·2	93·4	0·60	1·00			
Lunch	48·9	9·0	0	44·4	49·7	54·4	73·0	0·56	0·31	1·00		
Dinner	53·0	8·0	18·8	47·7	53·0	58·3	76·6	0·65	0·33	0·17	1·00	
Snacks	34·2	15·2	0	26·4	35·3	43·3	78·1	0·29	0·16	0·09	0·14	1·00
NRF9.3†												
Total diet	667	106	142	611	679	743	884	1·00				
Breakfast	556	225	−1264	478	599	700	870	0·51	1·00			
Lunch	598	149	−45	509	619	704	890	0·58	0·18	1·00		
Dinner	665	110	51	604	683	742	882	0·70	0·24	0·25	1·00	
Snacks	98	383	−1948	−28	123	351	799	0·34	0·16	0·15	0·17	1·00

Q25, 25th percentile; Q75, 75th percentile.

* A maximum score is 100. A higher score indicates a higher diet quality.

† A maximum score is 900. A higher score indicates a higher diet quality.

Table 2 shows total scores of the HEI-2015 and NRF9.3 according to each category of basic characteristics. Compared with men, women had a higher mean value of HEI-2015 for breakfast and lunch but a lower mean value of NRF9.3 for total diet. There was a positive association of age with all diet quality variables, while there was no association for weight status. Participants identified as underreporters had lower diet quality scores than those identified as plausible reporters, overreporters or both, except for NRF9.3 for breakfast and snacks. Participants in the 2013 survey had lower diet quality scores than those in the 2003 survey, except for NRF9.3 for lunch.

**Table 2:** Total scores of the Healthy Eating Index-2015 (HEI-2015) and Nutrient-Rich Food Index 9.3 (NRF9.3) according to each category of basic characteristics in 639 Japanese adults aged 20–81 years

	*n*	HEI-2015*										NRF9.3†									
		Total diet		Breakfast		Lunch		Dinner		Snacks		Total diet		Breakfast		Lunch		Dinner		Snacks	
		Mean	sd	Mean	sd	Mean	sd	Mean	sd	Mean	sd	Mean	sd	Mean	sd	Mean	sd	Mean	sd	Mean	sd
Sex																					
Male	318	51·7	7·3	44·0	12·1	48·2	9·2	53·0	8·3	33·5	16·3	678	104	563	230	597	151	667	111	93	438
Female	321	52·5	7·4	46·1	12·8	49·6	8·7	53·0	7·6	34·9	14·1	657	107	549	220	598	147	662	110	103	321
*P* ‡		0·21		0·04		0·0497		0·96		0·23		0·01		0·46		0·92		0·62		0·75	
Age group (years)																					
<40	205	48·2^a^	6·5	40·4^a^	12·2	46·3^a^	9·3	49·7^a^	7·5	30·7^a^	14·5	615^a^	114	474^a^	259	569^a^	150	622^a^	117	5^a^	410
40–59	279	52·5^b^	6·6	45·2^b^	11·2	48·9^b^	8·8	53·5^b^	7·7	35·2^b^	15·0	675^b^	92	558^b^	211	600^ab^	143	671^b^	103	130^b^	369
≥60	155	56·6^c^	6·9	50·9^c^	12·7	52·4^c^	7·9	56·6^c^	7·4	37·0^b^	15·8	723^c^	84	661^c^	141	632^b^	152	709^c^	92	163^b^	351
*P* §		<0·0001		<0·0001		<0·0001		<0·0001		0·0001		<0·0001		<0·0001		0·0003		<0·0001		<0·0001	
Weight status||																					
Underweight	35	50·5	6·2	45·0	11·1	47·3	8·2	52·0	7·6	35·1	13·3	633	117	549	234	582	130	627^a^	111	79	344
Normal weight	445	52·1	7·2	45·0	12·8	48·8	9·0	52·9	7·9	33·9	15·1	671	101	552	233	599	150	670^a^	110	97	370
Overweight	159	52·5	7·9	45·2	12·0	49·5	9·3	53·6	8·2	34·9	16·0	664	116	569	198	598	152	656^a^	110	103	427
*P* §		0·33		0·98		0·40		0·44		0·73		0·10		0·71		0·80		0·047		0·95	
Dietary reporting status¶																					
Underreporting	20	46·8^a^	9·4	36·9^a^	16·4	41·1^a^	16·9	48·2^a^	9·6	24·7^a^	15·5	605^a^	150	463	215	503^a^	236	585^a^	171	−99^a^	529
Plausible reporting	604	52·2^b^	7·2	45·2^b^	12·3	49·1^b^	8·5	53·1^b^	7·9	34·5^b^	15·0	671^b^	102	560	225	601^b^	145	669^b^	106	106^a^	371
Overreporting	15	54·9^b^	7·9	47·5^b^	12·0	49·4^b^	9·9	55·7^b^	8·5	36·4^ab^	19·0	615^ab^	159	521	221	589^ab^	156	597^a^	132	25^a^	566
*P* §		0·002		0·01		0·0004		0·01		0·02		0·004		0·14		0·01		0·0002		0·047	
Survey year																					
2003	250	53·6	6·9	47·0	11·9	49·9	8·5	54·9	8·1	35·7	14·9	689	96	586	225	601	147	684	97	136	326
2013	389	51·2	7·5	43·8	12·7	48·3	9·3	51·8	7·7	33·2	15·3	653	110	537	223	595	151	652	116	73	414
*P* ‡		<0·0001		0·001		0·03		<0·0001		0·04		<0·0001		0·006		0·62		0·0003		0·04	

* A maximum score is 100. A higher score indicates a higher diet quality.

† A maximum score is 900. A higher score indicates a higher diet quality.

‡ Based on independent *t* test.

§ Based on ANOVA. When the overall *P* from ANOVA was < 0·05, a Bonferroni’s *post hoc* test was performed; mean values within each variable with unlike superscript letters are significantly different (*P* < 0·05).

|| Underweight, normal weight and overweight were defined as participants having a BMI (in kg/m^2^) of <18·5, ≥18·5 to <25 and ≥25, respectively.

¶ Underreporting, plausible reporting and overreporting were defined as participants having a ratio of reported energy intake:BMR of <1·02, ≥1·02 to <2·35 and ≥2·35, respectively.

Breakfast, lunch, dinner and snacks, on average, accounted for 21, 32, 40 and 11 % of total EI, respectively (Table 3). For nutrients, the percentage contribution to total intake tended to be similar in magnitude to that for EI: 18–24 % for breakfast, except for alcohol (4 %) and Ca (29 %); 26–35 % for lunch, except for alcohol (20 %); 35–49 % for dinner, except for added sugars (27 %) and alcohol (70 %); and 4–15 % for snacks, except for added sugars (37 %). Breakfast, lunch and dinner were relatively similar in terms of percentage of energy from macronutrients, while characteristics of snacks included high intakes of saturated fats and added sugars and low intakes of protein and total fat.

**Table 3:** Intakes of energy and nutrients from each meal and their percentage contribution to total intake in 639 Japanese adults aged 20–81 years*

			Amount consumed								Contribution to total diet (%)||							
	Total diet		Breakfast†		Lunch‡		Dinner		Snacks§		Breakfast		Lunch		Dinner		Snacks	
	Mean	sd	Mean	sd	Mean	sd	Mean	sd	Mean	sd	Mean	sd	Mean	sd	Mean	sd	Mean	sd
Energy (kJ/d)	8774	2028	1838	774	2737	746	3518	1211	962	840	21·1	8·0	31·6	7·3	40·0	9·4	10·9	8·5
Nutrients																		
Protein (g/d)	74·4	18·3	15·6	7·8	22·8	7·1	33·0	11·4	4·9	4·6	21·0	9·3	31·1	8·6	44·2	10·2	6·6	5·7
Total fat (g/d)	64·6	19·2	13·3	7·7	19·3	7·7	27·0	11·8	7·2	8·0	20·7	10·4	30·5	10·9	41·5	12·3	10·9	10·8
SFA (g/d)	18·7	6·3	4·5	2·9	5·0	2·4	7·0	3·5	3·0	3·3	23·9	12·8	27·6	12·3	37·5	13·5	15·3	15·0
Carbohydrate (g/d)	278·0	68·5	63·4	27·6	93·8	26·6	97·5	33·2	32·5	25·7	22·8	8·3	34·1	7·5	35·1	8·9	11·5	8·3
Added sugars (g/d)	32·6	19·1	6·2	7·2	8·1	7·3	7·9	6·9	12·6	12·0	18·5	16·3	26·1	16·7	26·5	17·3	36·8	29·9
Alcohol (g/d)	11·4	19·9	0·1	0·7	0·5	2·5	9·1	17·3	2·3	10·0	4·0	13·7	19·5	30·3	69·7	35·9	10·3	32·6
Dietary fibre (g/d)	14·3	4·8	3·2	2·0	4·4	1·9	5·9	2·4	1·2	1·4	21·8	10·1	31·4	9·8	42·1	11·6	7·9	8·5
Na (mg/d)	4143	1157	843	511	1446	541	1794	697	150	188	20·2	11·0	35·3	10·1	43·3	11·2	3·7	4·6
K (mg/d)	2687	762	591	335	744	284	1123	398	304	257	21·6	9·3	28·1	8·8	42·4	10·9	11·3	8·6
Ca (mg/d)	533·5	193·3	161·3	114·4	133·8	59·8	186·5	88·5	69·9	71·1	28·8	14·5	26·3	10·7	36·0	13·0	13·2	11·9
Mg (mg/d)	295·5	88·7	65·3	37·0	80·5	29·7	127·7	53·2	30·0	28·1	21·9	9·6	27·9	8·5	43·4	11·4	10·2	8·1
Fe (mg/d)	8·4	2·3	1·8	1·1	2·5	0·8	3·6	1·4	0·8	0·7	20·7	10·5	30·6	8·9	42·9	11·5	9·1	7·6
Vitamin A (μg/d)¶	616·7	678·2	116·6	231·4	183·5	261·2	291·2	550·3	38·8	62·3	20·5	14·0	31·8	17·0	43·3	18·1	7·5	9·9
Vitamin D (mg/d)	7·9	5·5	1·5	2·4	2·2	2·3	4·1	4·1	0·2	0·4	19·9	19·9	30·4	24·7	48·7	25·7	4·0	7·8
Vitamin C (mg/d)	112·7	52·5	22·3	21·6	33·4	18·8	48·0	26·1	11·6	16·4	18·4	12·9	31·2	14·7	44·1	16·2	9·5	12·4
Percentage of energy																		
Protein	14·3	2·1	14·0	3·9	14·0	2·8	16·1	3·6	8·7	6·3	–	–	–	–	–	–	–	–
Total fat	27·8	5·2	26·2	10·4	26·2	7·0	28·9	7·6	23·3	15·6	–	–	–	–	–	–	–	–
SFA	8·1	2·1	9·1	4·8	6·8	2·7	7·5	2·6	10·0	7·7	–	–	–	–	–	–	–	–
Carbohydrate	53·3	6·6	58·3	12·0	57·4	8·3	47·6	10·3	55·5	23·8	–	–	–	–	–	–	–	–
Added sugars	6·2	3·2	6·2	8·3	4·9	3·9	3·8	2·9	21·8	17·8	–	–	–	–	–	–	–	–
Alcohol	3·4	5·7	0·1	0·5	0·4	1·5	6·0	10·0	4·0	12·9	–	–	–	–	–	–	–	–

* Values are per capita intakes unless otherwise indicated.

† For the calculation of percentage of energy, *n* 634 after excluding five non-breakfast consumers.

‡ For the calculation of percentage of energy, *n* 636 after excluding three non-lunch consumers.

§ For the calculation of percentage of energy, *n* 593 after excluding forty-six non-snack consumers.

|| For alcohol, *n* 615 because there were twenty-four participants whose total alcohol intake was zero.

¶ Retinol activity equivalent.

However, intakes of many food groups were largely dependent on one meal type (Table 4). The foods mainly eaten at dinner were potatoes, pulses, total vegetables, fish, meat and alcoholic beverages (52–70 %), in contrast to noodles (58 %) at lunch and bread (71 %) and dairy products (50 %) at breakfast. The foods mainly eaten at snacks were confectioneries (79 %) and sugar-sweetened beverages (52 %). Conversely, intakes of rice and eggs were more evenly distributed across three main meals (19–41 % and 30–38 %, respectively). Additionally, intakes of fruit and non-energetic beverages were more evenly distributed across all meal types (17–30 % and 19–35 %, respectively).

**Table 4:** Intakes of foods from each meal and their percentage contribution to total intake in 639 Japanese adults aged 20–81 years*

	Amount consumed (g/d)										*n* †	Contribution to total diet (%)‡							
	Total diet		Breakfast		Lunch		Dinner		Snacks			Breakfast		Lunch		Dinner		Snacks	
	Mean	sd	Mean	sd	Mean	sd	Mean	sd	Mean	sd		Mean	sd	Mean	sd	Mean	sd	Mean	sd
Rice	327·5	143·2	66·9	70·1	131·9	71·6	133·7	73·6	1·5	9·3	638	19·4	19·3	41·2	23·1	41·4	17·9	0·6	3·7
Bread	38·1	33·4	28·2	29·9	7·4	16·0	1·8	6·9	2·0	7·4	503	70·7	40·8	21·1	38·2	5·7	19·1	6·1	21·0
Noodles	75·0	66·3	4·1	20·0	43·9	50·9	27·3	42·6	1·1	8·6	533	5·0	18·3	57·6	40·7	37·5	39·4	1·2	9·1
Potatoes	44·2	36·0	4·9	10·8	12·9	17·3	25·0	26·3	2·2	8·7	622	10·3	20·5	31·2	30·8	56·0	33·1	4·6	15·5
Pulses§	67·0	52·6	16·1	26·9	14·1	19·5	34·2	34·6	4·0	13·9	630	22·9	27·3	22·6	24·2	51·8	31·2	5·1	14·9
Total vegetables	264·5	116·2	39·7	42·1	83·1	50·9	142·5	74·5	3·1	13·1	639	13·9	12·7	32·1	16·4	54·8	17·6	1·1	4·2
Fruit	70·4	73·9	24·4	38·5	17·3	26·8	17·2	31·1	13·4	28·6	589	29·1	33·4	30·4	34·7	26·8	33·2	17·3	31·7
Fish||	75·7	47·6	8·0	14·3	22·6	20·3	45·3	34·8	1·0	5·5	634	9·4	15·7	33·4	30·4	58·4	27·3	1·2	5·7
Meat	84·0	50·5	6·7	11·7	27·6	22·5	50·2	36·6	1·0	4·3	636	7·5	11·1	33·5	22·7	59·6	23·4	1·4	7·7
Eggs	39·5	22·5	13·6	15·3	14·2	14·0	11·5	12·8	1·0	3·1	632	32·5	32·2	37·6	31·1	29·5	27·9	3·0	10·1
Dairy products	115·6	103·7	65·4	76·9	17·0	35·1	14·6	29·3	23·3	45·0	614	50·4	38·4	18·8	32·6	15·5	26·0	23·0	41·6
Confectioneries	38·8	33·7	4·7	12·0	5·8	14·3	4·7	11·5	28·3	31·8	552	11·5	25·8	13·6	26·0	12·6	26·6	78·7	66·0
Alcoholic beverages	146·3	255·6	0·4	2·5	6·3	44·4	118·0	222·0	28·0	112·4	589	3·4	12·4	19·4	30·7	70·3	36·2	10·3	31·9
Sugar-sweetened beverages	33·2	72·6	5·6	25·7	6·3	25·0	8·0	42·5	15·5	45·1	243	18·4	35·6	21·2	39·7	18·2	36·2	51·9	65·8
Nonenergetic beverages	753·3	480·6	185·1	151·3	172·8	125·6	139·2	122·1	289·1	359·7	634	25·2	19·7	25·5	17·7	19·4	16·2	35·3	27·1

* Values are per capita intakes unless otherwise indicated.

† Number of consumers.

‡ Calculated based on consumers only.

§ Including nuts.

|| Including shellfish.

EI from breakfast (but not lunch or dinner) was positively associated with overall diet quality as assessed by total scores of HEI-2015 (Table 5) and NRF9.3 (data not shown) of total diet. Conversely, associations with overall diet quality were relatively consistent for intakes of nutrients across three main meals. A higher overall diet quality was associated with higher intakes of protein, dietary fibre, K, Ca, Mg, Fe and vitamin C from all three main meals. In terms of food groups, a higher overall diet quality was associated with higher intakes of pulses, total vegetables and fish from all three main meals (Table 6 for HEI-2015 and data not shown for NRF9.3). Additionally, a higher overall diet quality was associated with higher intakes of rice, eggs and dairy products from breakfast, potatoes from lunch, and fruit from breakfast and dinner and lower intakes of bread from breakfast and lunch, confectioneries from lunch and sugar-sweetened beverages from dinner. Associations between intakes from snacks and overall diet quality were relatively weak in magnitude, notwithstanding an inverse association between snack energy and the NRF9.3 score (data not shown).

**Table 5:** Associations of intakes of energy and nutrients from breakfast, lunch and dinner with total scores of the Healthy Eating Index-2015 (HEI-2015) of total diet in 639 Japanese adults aged 20–81 years*

	Breakfast†						*P*_for trend_	Lunch‡						*P*_for trend_	Dinner						*P*_for trend_
	T1 (*n* 213)		T2 (*n* 213)		T3 (*n* 213)			T1 (*n* 213)		T2 (*n* 213)		T3 (*n* 213)			T1 (*n* 213)		T2 (*n* 213)		T3 (*n* 213)		
	Mean	se	Mean	se	Mean	se		Mean	se	Mean	se	Mean	se		Mean	se	Mean	se	Mean	se	
Energy (kJ/d)	1745	52	1857	50	1913	52	0·03	2714	46	2803	44	2695	47	0·80	3404	68	3640	65	3511	69	0·28
Nutrients																					
Protein (g/d)	13·9	0·5	15·6	0·5	17·4	0·5	< 0·0001	21·7	0·5	23·2	0·4	23·4	0·5	0·01	31·2	0·7	33·7	0·7	34·0	0·7	0·008
Total fat (g/d)	13·5	0·5	13·0	0·5	13·4	0·5	0·87	19·6	0·5	19·4	0·5	18·8	0·5	0·32	26·5	0·8	27·9	0·8	26·6	0·8	0·93
SFA (g/d)	4·8	0·2	4·3	0·2	4·3	0·2	0·07	5·4	0·2	5·1	0·2	4·6	0·2	0·001	7·2	0·2	7·4	0·2	6·4	0·2	0·04
Carbohydrate (g/d)	58·9	1·9	64·9	1·8	66·4	1·9	0·006	92·7	1·7	96·8	1·6	91·9	1·7	0·79	98·5	2·0	100·7	1·9	93·2	2·0	0·09
Added sugars (g/d)	6·6	0·5	5·6	0·5	6·3	0·5	0·63	8·9	0·5	8·0	0·5	7·4	0·5	0·051	7·8	0·5	8·5	0·5	7·4	0·5	0·61
Alcohol (g/d)	0·0	0·0	0·1	0·0	0·1	0·0	0·56	0·3	0·2	0·5	0·2	0·6	0·2	0·40	5·9	1·1	9·6	1·1	11·7	1·1	0·0006
Dietary fibre (g/d)	2·6	0·1	3·1	0·1	3·9	0·1	< 0·0001	3·9	0·1	4·3	0·1	4·9	0·1	< 0·0001	5·0	0·2	6·0	0·2	6·7	0·2	< 0·0001
Na (mg/d)	778	34	872	33	879	35	0·047	1457	37	1479	35	1402	37	0·32	1817	47	1834	45	1730	47	0·22
K (mg/d)	462	20	555	19	756	20	< 0·0001	668	19	756	19	809	20	< 0·0001	994	26	1140	25	1234	26	< 0·0001
Ca (mg/d)	131·2	7·7	149·3	7·4	203·4	7·7	< 0·0001	121·2	4·1	133·6	4·0	146·7	4·2	< 0·0001	162·6	6·0	194·1	5·8	202·9	6·1	< 0·0001
Mg (mg/d)	52·8	2·3	62·1	2·2	81·1	2·3	< 0·0001	72·4	2·0	80·0	1·9	89·2	2·0	< 0·0001	112·1	3·5	131·2	3·4	139·9	3·5	< 0·0001
Fe (mg/d)	1·4	0·1	1·7	0·1	2·2	0·1	< 0·0001	2·3	0·1	2·5	0·1	2·7	0·1	< 0·0001	3·3	0·1	3·6	0·1	3·8	0·1	< 0·0001
Vitamin A (μg/d)§	79·9	16·5	123·9	15·8	145·8	16·6	0·007	158·6	18·5	189·9	17·8	201·8	18·7	0·11	251·5	39·4	299·9	37·8	322·2	39·7	0·23
Vitamin D (mg/d)	1·3	0·2	1·5	0·2	1·8	0·2	0·04	1·9	0·2	2·3	0·2	2·3	0·2	0·16	3·6	0·3	4·2	0·3	4·6	0·3	0·02
Vitamin C (mg/d)	14·6	1·4	20·7	1·3	31·6	1·4	< 0·0001	27·4	1·3	32·7	1·2	40·3	1·3	< 0·0001	40·6	1·8	47·1	1·7	56·3	1·8	< 0·0001
Percentage of energy																					
Protein	13·1	0·3	14·0	0·3	15·0	0·3	< 0·0001	13·4	0·2	13·9	0·2	14·6	0·2	< 0·0001	15·6	0·2	16·0	0·2	16·6	0·2	0·009
Total fat	27·8	0·7	25·5	0·7	25·2	0·7	0·02	26·8	0·5	25·6	0·5	26·1	0·5	0·38	29·4	0·5	28·9	0·5	28·4	0·5	0·18
SFA	10·3	0·3	8·7	0·3	8·3	0·3	< 0·0001	7·4	0·2	6·7	0·2	6·4	0·2	0·0004	7·9	0·2	7·6	0·2	6·9	0·2	0·0001
Carbohydrate	57·0	0·9	59·1	0·8	58·8	0·9	0·16	56·9	0·6	57·8	0·6	57·4	0·6	0·57	48·7	0·7	47·6	0·7	46·5	0·7	0·04
Added sugars	7·3	0·6	5·4	0·6	5·9	0·6	0·11	5·3	0·3	4·6	0·3	4·6	0·3	0·07	3·9	0·2	3·8	0·2	3·7	0·2	0·60
Alcohol	0·0	0·0	0·1	0·0	0·1	0·0	0·52	0·3	0·1	0·4	0·1	0·5	0·1	0·36	4·4	0·7	6·0	0·6	7·5	0·7	0·002

T, tertile.

* Examined using the general linear model, with adjustment for sex, age group, weight status, dietary reporting status and survey year. Median values (ranges) of total scores of the HEI-2015 of total diet for the first, second and third tertile categories were 45·7 (22·1–48·3), 51·3 (48·4–55·1) and 59·3 (55·2–77·2), respectively. A higher score indicates a higher diet quality.

† For variables expressed as percentage of energy, *n* 634 after excluding five non-breakfast consumers.

‡ For variables expressed as percentage of energy, *n* 636 after excluding three non-lunch consumers.

§ Retinol activity equivalent.

**Table 6:** Associations of intakes of foods from breakfast, lunch and dinner with total scores of the Healthy Eating Index-2015 (HEI-2015) of total diet in 639 Japanese adults aged 20–81 years*

	Breakfast (g/d)						*P*_for trend_	Lunch (g/d)						*P*_for trend_	Dinner (g/d)						*P*_for trend_
	T1 (*n* 213)		T2 (*n* 213)		T3 (*n* 213)			T1 (*n* 213)		T2 (*n* 213)		T3 (*n* 213)			T1 (*n* 213)		T2 (*n* 213)		T3 (*n* 213)		
	Mean	se	Mean	se	Mean	se		Mean	se	Mean	se	Mean	se		Mean	se	Mean	se	Mean	se	
Rice	56·2	4·9	74·1	4·7	70·4	4·9	0·048	128·6	4·7	142·0	4·5	125·1	4·7	0·64	142·9	4·9	141·3	4·7	117·0	4·9	0·0004
Bread	35·2	2·1	29·0	2·0	20·4	2·1	< 0·0001	9·8	1·1	7·4	1·1	5·0	1·1	0·0048	2·2	0·5	2·1	0·5	1·1	0·5	0·11
Noodles	6·3	1·4	4·7	1·4	1·2	1·4	0·01	46·5	3·6	40·3	3·5	45·0	3·7	0·77	32·8	2·9	23·6	2·8	25·6	3·0	0·09
Potatoes	3·2	0·7	4·8	0·7	6·7	0·8	0·002	11·1	1·2	12·8	1·2	14·8	1·2	0·04	22·7	1·9	25·0	1·8	27·2	1·9	0·11
Pulses†	9·9	1·8	14·2	1·8	24·2	1·8	< 0·0001	11·6	1·4	13·7	1·3	17·1	1·4	0·007	28·8	2·4	35·3	2·4	38·5	2·5	0·007
Total vegetables	27·7	2·6	39·2	2·5	52·2	2·7	< 0·0001	72·9	3·6	83·1	3·4	93·2	3·6	0·0001	120·5	5·1	147·3	4·9	159·7	5·2	< 0·0001
Fruit	10·0	2·5	22·0	2·4	41·3	2·6	< 0·0001	10·1	1·8	15·7	1·7	26·2	1·8	< 0·0001	7·5	2·0	13·9	2·0	30·2	2·1	< 0·0001
Fish‡	5·2	1·0	7·4	0·9	11·4	1·0	< 0·0001	17·6	1·4	24·1	1·3	25·9	1·4	< 0·0001	39·3	2·4	46·5	2·3	50·2	2·4	0·002
Meat	7·0	0·8	7·5	0·8	5·5	0·8	0·19	28·6	1·5	27·5	1·5	26·8	1·5	0·42	50·2	2·4	51·6	2·3	48·8	2·5	0·70
Eggs	11·3	1·1	14·6	1·0	15·0	1·1	0·02	15·1	1·0	14·1	1·0	13·6	1·0	0·32	11·6	0·9	12·0	0·9	10·9	0·9	0·60
Dairy products	53·7	5·4	59·5	5·2	83·0	5·4	0·0003	14·1	2·5	18·4	2·4	18·4	2·5	0·24	13·6	2·1	14·4	2·0	15·9	2·1	0·47
Confectioneries	5·3	0·9	4·8	0·8	4·0	0·9	0·30	8·1	1·0	4·9	1·0	4·4	1·0	0·01	4·4	0·8	5·8	0·8	4·0	0·8	0·75
Alcoholic beverages	0·2	0·2	0·7	0·2	0·4	0·2	0·44	3·7	3·2	6·7	3·0	8·5	3·2	0·31	83·8	14·9	129·9	14·3	140·1	15·0	0·01
Sugar-sweetened beverages	7·6	1·8	5·3	1·8	4·0	1·9	0·19	7·3	1·8	8·0	1·7	3·5	1·8	0·16	13·3	3·0	7·7	2·9	2·9	3·0	0·02
Nonenergetic beverages	200·0	10·7	173·6	10·3	181·9	10·8	0·25	175·9	9·0	161·4	8·6	181·2	9·0	0·71	154·5	8·6	130·6	8·3	132·4	8·7	0·08

T, tertile.

* Examined using the general linear model, with adjustment for sex, age group, weight status, dietary reporting status and survey year. Median values (ranges) of total scores of the HEI-2015 of total diet for the first, second and third tertile categories were 45·7 (22·1–48·3), 51·3 (48·4–55·1) and 59·3 (55·2–77·2), respectively. A higher score indicates a higher diet quality.

† Including nuts.

‡ Including shellfish.

## Discussion

In this study of Japanese adults, diet quality, which was assessed by the HEI-2015 and NRF9.3, was highest for dinner, followed by lunch, breakfast and snacks. The order was in line with the percentage contribution of each meal type to total EI and intakes of many nutrients. However, dinner was mainly characterised by the intake of potatoes, pulses, total vegetables, fish, meat and alcoholic beverages; lunch by noodles; breakfast by bread and dairy products; and snacks by confectioneries and sugar-sweetened beverages. Conversely, intakes of rice and eggs were more evenly distributed across three main meals, while intakes of fruit and non-energetic beverages were more evenly distributed across all four meal types. We further identified dietary intake patterns within each meal type associated with a higher quality of total diet. To our knowledge, this is the first study to characterise different meal types in Japanese adults, which would make an important contribution to the existing literature of meal intakes and patterns^(5–9)^.

In this study, the mean percentage contribution to total EI was 21 % for breakfast, 32 % for lunch, 40 % for dinner and 11 % for snacks. This is consistent with that observed in the National Health and Nutrition Survey in Japan^(23)^, but is quite different from that in Western populations, where, generally speaking, the percentage contribution of breakfast is smaller and that of snacks is larger^(24,25)^. Thus, in relation to EI, the Japanese meal patterns may be characterised by three main meals, accompanied by small contribution from snacks in terms of both size and frequency. It has been suggested that consuming a high proportion of total EI at main meals (particularly lunch), as well as a small contribution of snacks, is an additional positive component of the Mediterranean diet, aside from the solely nutritional considerations^(25,50)^. Thus, these characteristics we observed, in addition to the very low prevalence of meal skipping and the well-structured meal timing, might contribute to the health effect of Japanese diets, if any, in addition to the amount and content of food and nutrient intake. Further research on this topic is warranted.

We found that for many of the macro- and micronutrients, the percentage contribution to total intake did not vary within each meal type and tended to be similar to that for EI, implying that the nutrient density of each meal type is similar in Japanese dietary habits. This is generally consistent with findings from previous Western studies, at least for macronutrients^(7–9)^. However, in contrast to our findings, evidence from several Western countries, including Canada, Denmark, France, Spain, the UK and the USA^(51)^, suggests that for micronutrients, the percentage contribution to total intake did vary, especially at breakfast. The exact reason is unknown, but this might be due to differences in the degree of dependence on one meal type for key foods, particularly plant-based foods. For example, a study in Norwegian adults showed that dinner accounted for 69 % (of daily intake) of vegetables (and 72 % of fish), breakfast accounted for 44 % of whole grains and snacks accounted for 51 % of fruits^(5)^. Similar findings were observed from a national representative sample of Australian adults for non-starchy and starchy vegetables (62 and 81 % from dinner, respectively) and fruits (55 % from snacks)^(6)^. In contrast, intakes of major plant-based foods were more evenly distributed among three main meals in this study: the contribution of breakfast, lunch and dinner was, respectively, 14, 32 and 55 % for total vegetables; 29, 30 and 27 % for fruit (as well as 17 % from snacks) and 19, 41 and 41 % for rice (the major grain food). In any case, this kind of background information should be accumulated from various countries so that more effective country-specific meal-based dietary guidelines and public health messages could be developed.

We found that favourable dietary intake patterns associated with a higher diet quality of total diet were relatively consistent across three main meal types. That is, there was a positive association of overall diet quality with intakes of key nutrients and food groups to encourage from each main meal. However, one clear exception to note was rice. A higher intake of rice from breakfast was positively associated with a higher overall diet quality, but such a positive association was not observed for lunch or dinner. This may be because there are multiple options for staple foods (rice or bread) in breakfast in Japan^(22,31)^, and thus, the association with overall diet quality may be assessed mainly as a comparison of a choice of rice or bread within breakfast. For support on this, bread intake at breakfast was inversely associated with overall diet quality. On the other hand, because rice is more exclusively selected as a staple food for lunch and dinner^(22,31)^, the association with overall diet quality may be assessed mainly as a comparison of other foods accompanied by rice within lunch and dinner. These observations, as well as relatively weak correlations of diet quality among meal types, highlight the complex nature of meals as food combinations, which, in turn, suggests the importance of accumulating evidence at the meal level.

The strength of this study is the use of detailed dietary information obtained from a 4-d weighed dietary record. However, there are also several limitations. First, although sampling was conducted to consider regional differences in dietary habits, the present population is not a nationally representative sample of general Japanese, but rather volunteers, of whom some lived in the same household. In particular, our participants may be biased towards greater health consciousness. We unfortunately do not know how the present population is comparable with the general Japanese population in terms of, for example, educational level and employment because of a lack of information. Nevertheless, the mean values of the HEI-2015 in the present population were comparable with those reported from the 2012 Japanese National Health and Nutrition Survey (51·3 (sd 9·0) for men and 52·9 (sd 9·2) for women; information not available for NRF9.3)^(18)^. Further research in a more representative sample is needed.

Second, all self-reported dietary assessment methods are subject to both random and systematic errors^(52)^, and the nature and extent of the measurement error of self-reported information on dietary intake from each meal type are largely unknown^(24)^. The present results should therefore be interpreted with caution in this respect. Nevertheless, the use of the Goldberg’s cut-off (based on EI:BMR) identified only a few underreporters and overreporters (3·1 and 2·3 %, respectively), suggesting overall satisfactory reporting accuracy of dietary intake. To minimise the influence of measurement error in dietary variables, we mainly relied on the percentage contribution to total intake for interpreting our data, as well as the use of energy-adjusted values for diet quality measures^(53)^.

Third, because the dietary data used here were collected during the winter season (February and March), any seasonal variation in dietary intake was not considered. Given that several previous studies have observed seasonal differences in intakes of at least some nutrients and food groups in Japanese adults^(54–56)^, this might have produced some bias in assessing average dietary intake over the year. Additionally, while two independent data collected in 2003 and 2013 were combined for the main analysis after confirming that separate analyses produced similar results, this 10-year difference might be potentially significant when it comes to diet. Given that a trend analysis based on National Health and Nutrition Survey 2003–2015^(14)^ showed significant albeit small differences in food group intakes during this period, the present results should be interpreted cautiously.

Fourth, diet quality was assessed by the HEI-2015 and NRF9.3 in this Japanese study, even though both scores were primarily developed for Americans. Thus, these measures are not optimal for assessing the overall quality of Japanese diet, but rather the best available^(18)^, as mentioned above. Finally, in view of the multiple analyses for the associations between overall diet quality and intakes from each meal type, it is possible that some of the significant findings in the present study occurred by chance.

In conclusion, in this study of Japanese adults, dinner was highest in terms of not only diet quality but also percentage contribution to total EI, followed by lunch, breakfast and snacks. For many nutrients, the percentage contribution to total intake did not vary across each meal type and was broadly in line with that for EI. For foods, on the other hand, intakes largely depended on one meal type, except for several foods, including rice, eggs, fruit and non-energetic beverages, which were more commonly consumed at various meal types. This study also provided a picture of dietary intake patterns within each meal type associated with a higher quality of total diet. These findings provide the key information on each meal type in the Japanese diet and will help inform the development of meal-based guidelines and public health messages. Future research is needed to examine whether the present findings are similarly observed in a more representative sample of Japanese as well as in other Asian populations.

## Supplementary material

For supplementary material accompanying this paper visit https://doi.org/10.1017/S1368980020004310. click here to view supplementary material
