# Supplementary material for: Characterisation of breakfast, lunch, dinner and snacks in the Japanese context: an exploratory cross-sectional analysis
Source: Public Health Nutr. 2020 Nov 10;25(3):689–701. doi: 10.1017/S1368980020004310 (PMC9991704; doi:10.1017/S1368980020004310)
Supplement: Supplementary file 1 [file S1368980020004310sup001.docx]

Supplemental Table 1. Number and proportion of participants by number of reporting days of consumption of breakfast, lunch, dinner, and snack in 639 Japanese adults aged 20-81 years

|  | Breakfast | | Lunch | | Dinner | | Snack* | |
| --- | --- | --- | --- | --- | --- | --- | --- | --- |
| Number of days | *n* | % | *n* | % | *n* | % | *n* | % |
| 0 | 5 | 0.8 | 3 | 0.5 | 0 | 0 | 46 | 7.2 |
| 1 | 4 | 0.6 | 2 | 0.3 | 0 | 0 | 44 | 6.9 |
| 2 | 16 | 2.5 | 3 | 0.5 | 2 | 0.3 | 56 | 8.8 |
| 3 | 52 | 8.1 | 41 | 6.4 | 18 | 2.8 | 110 | 17.2 |
| 4 | 562 | 88.0 | 590 | 92.3 | 619 | 96.9 | 383 | 59.9 |

* Reporting at least one snack.
